# Precision Medicine Advances in Chronic Lung Diseases

**DOI:** 10.3390/ijms262311243

**Published:** 2025-11-21

**Authors:** Agamemnon Bakakos, Vasilios Tzilas, Stelios Loukides, Petros Bakakos, Theodoros Karampitsakos

**Affiliations:** 1 1st Department of Respiratory Medicine, National and Kapodistrian University of Athens, 11527 Athens, Greece; agabak@med.uoa.gr (A.B.); petros44@hotmail.com (P.B.); 2 2nd Department of Respiratory Medicine, National and Kapodistrian University of Athens, 12462 Athens, Greece; tzilasvasilios@gmail.com (V.T.); loukstel@med.uoa.gr (S.L.)

## 1. Introduction

During the last few years, we have witnessed monumental scientific advancements in our knowledge of the pathogenic drivers of chronic lung diseases. Among others, genetics, epigenetics, immune aberrations, metabolic abnormalities, alterations in epithelial cell homeostasis and extracellular matrix remodeling have been implicated in the pathogenesis of various lung disorders. Examples of such pathogenic determinants of particular lung diseases are discussed in the articles included in the Special Issue entitled ‘Molecular Pathophysiology of Lung Diseases’. These pathogenic perturbations could be disease-specific, but, importantly, may only be present in a subset of patients with a specific disease. Acknowledgement of this observation is paving the way towards the personalized management of patients based on their pathogenetic background, given that a ‘one-size fits all approach’ in chronic lung diseases seems to be inappropriate. The articles featured in this Special Issue discuss the future aims of precision medicine application for common chronic lung diseases such as asthma and chronic obstructive lung disease (COPD) and less common chronic lung diseases such as interstitial lung diseases (ILDs) [1,2,3]. The aim of this Special Issue is to elucidate the complex inflammatory pathways altering the course of the aforementioned diseases and summarize novel treatment options on a tailored basis.

**Figure 1:**
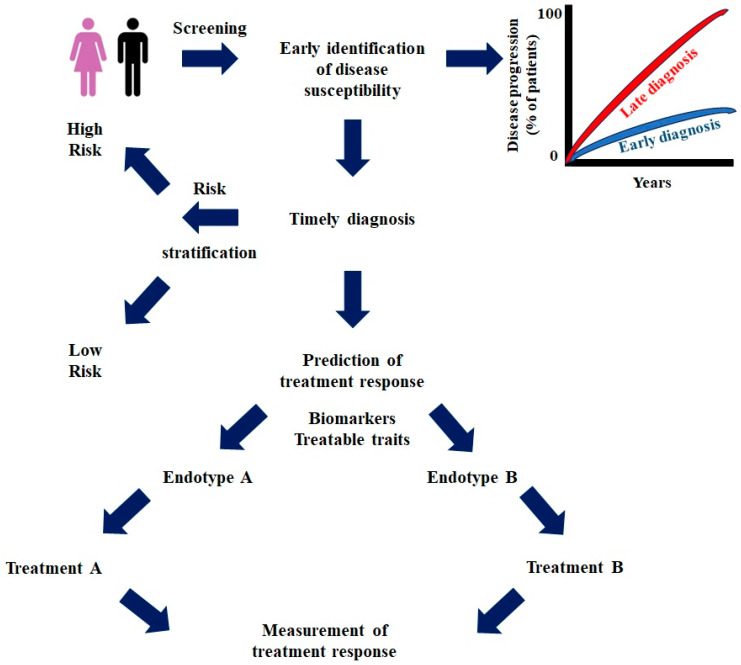
Schematic representation of a suggested roadmap to precision medicine in chronic lung diseases.

According to the U.S. Food and Drug Administration, precision medicine can be defined as “an innovative approach aiming to tailor disease prevention and treatment which takes into account differences in people’s genes, environments, and lifestyles. The aim of precision medicine is the implementation of the right treatment to the right patients at the right time [4].” Based on this definition, precision medicine or personalized medicine is directly associated with the following: (1) the use of biomarkers that are able to either identify disease susceptibility, timely diagnose diseases, predict disease progression and/or (2) the treatment regimens that could confer benefits to a subset of patients with a particular endotype (Figure 1). The latter could reduce unnecessary exposure to ineffective drugs and subsequently reduce the likelihood of side effects.

## 2. Lung Cancer

Precision medicine has already been implemented in chronic lung diseases, including lung cancer, asthma and COPD [5]. In particular, just in the past two years, more than ten targeted therapies have received approval for the management of non-small-cell lung cancer (NSCLC). The landscape of NSCLC has undergone a transformative evolution, where actionable genomic alterations (AGAs) serve as the cornerstone for precision oncology, allowing clinicians to select treatments according to the individual tumor characteristics identified in tissue biopsies [6,7]. Molecular diagnostics have become essential in guiding NSCLC management, with comprehensive genomic profiling identifying actionable mutations, such as *epidermal growth factor receptor (EGFR)*, *anaplastic lymphoma kinase (ALK)*, *ROS proto-oncogene 1 (ROS1)*, *Kirsten rat sarcoma viral oncogene homolog (KRAS)*, *B-Raf proto*-oncogene *(BRAF)*, *mesenchymal–epithelial transition factor receptor (MET)*, *rearranged during transfection (RET)*, *Neurotrophic tyrosine kinase receptor (NTRK-1/2/3)*, *Neuregulin 1 (NRG1)* and *Human Epidermal growth factor Receptor (HER2 or ERBB2)*. In the era of AGAs, circulating tumor DNA (ctDNA) is increasingly recognized as a valuable non-invasive diagnostic and predictive tool in monitoring NSCLC, offering a precision medicine approach that complements or even surpasses traditional tissue-based methods in select cases [8]. The beneficial results of precision medicine are exemplified by the integration of targeted therapies in early-stage disease management, with osimertinib for *EGFR* (+) NSCLC [9] and alectinib for *ALK* (+) disease [10], demonstrating substantial benefits in terms of progression-free survival (PFS) and overall survival (OS). Traditional chemotherapy-based approaches are becoming a less lucrative option compared to biologically informed treatment strategies [11].

Recent advances in lung cancer include the development of antibody–drug conjugates (ADCs), enabling the selective delivery of therapeutic agents to malignant cells while minimizing systemic toxicity, with trastuzumab deruxtecan becoming the first ever FDA-approved ADC for *HER2*-mutant NSCLC [7,12]. Other ADCs targeting trophoblast cell-surface antigen 2 (TROP2), HER3, mesenchymal–epithelial transition factor receptor tyrosine kinase (c-MET) and carcinoembryonic antigen-related cell adhesion molecule 5 (CECAM5) have shown promising results targeting molecular subtypes that were previously untreatable [12,13]. Bispecific antibodies constitute another innovative category, with the amivantamab–lazertinib combination being a representative in targeting *EGFR*-mutant NSCLC, including the challenging exon 20 insertion mutations and Osimertinib-pretreated advanced NSCLC [14]. Finally, biomarker-driven patient selection has enhanced the effectiveness of immune checkpoint inhibitors (ICIs), with programmed death-ligand 1 (PDL-1) expression levels serving as predictive markers for treatment response [15]. Immunotherapy has also been integrated in the preoperative management of lung cancer. More specifically, three cycles of neoadjuvant nivolumab plus chemotherapy conferred survival benefit in patients with resectable NSCLC as compared with chemotherapy alone [16].

## 3. Bronchial Asthma

In terms of bronchial asthma, precision medicine based on asthma endotypes has revolutionized our approach and demonstrated tremendous success in improving patients’ quality of life [17]. Since the implementation of a single monoclonal antibody (mAb) targeting immunoglobulin E (IgE)—omalizumab—in 2003 [18] and the addition of biologics targeting interleukin-5 (IL-5) almost fifteen years ago [19,20,21], the landscape for severe asthma has completely changed. In particular, there are currently many efficacious biologics for the T2-high endotype, leading to a significant reduction in oral corticosteroid use as maintenance treatment [20,22,23,24]. Currently, biologics target several pathways by blocking IgE, IL-5 and its receptor, IL-4R/IL-13R, and thymic stromal lymphopoietin (TSLP) [25]. Additional biologics are being evaluated in clinical trials, with promising results for biologics targeting IL-33 and other cytokines [26]. Despite such progress, the identification of treatable traits is an ongoing challenge in the management of severe asthma. Identification of asthma endotypes is currently based on underlying pathophysiological mechanisms and relies on the evaluation of several biomarkers, such as blood eosinophils, fractional exhaled nitric oxide, IgE levels and other less commonly used biomarkers such as sputum eosinophils, endobronchial tissue biopsies and periostin [27]. The combination of the aforementioned biomarkers with clinical features, such as the age of asthma onset, the presence of comorbidities such as chronic rhinosinusitis with nasal polyps, allergic rhinitis, urticaria and other diseases, is currently a standard procedure in expert asthma centers during the evaluation of a patient, in an effort to choose the right biologic. However, combining all the above does not guarantee accurate prediction of which biologic has the highest likelihood of leading to asthma remission [28]. Choosing the right biologic for the right patient is becoming increasingly challenging as there are no available head-to-head trials between them. Biologically enriched trials aimed at identifying the ideal biologic for the ideal endotype are greatly anticipated. In the meantime, thoughtful implementation is advisable. For example, even though long-term treatment with mAbs is generally considered effective and safe, a recent report that dupilumab is associated with an increased risk of T and natural killer (NK) lymphoma warrants caution and emphasizes that choosing between biologics is far more complex than it may seem [29]. Concerns have also arisen with regard to the risk of destructive polyarthritis following anti-IL-5/-5R treatment [30].

Although our understanding and the arsenal for T2-high asthma are becoming more advanced, this is not the case for non-T2-high asthma (neutrophilic, paucigranulocytic) [31]. Most of the existing biologics, with the exception of tezepelumab (which blocks TSLP), are ineffective in T2-low asthma; even tezepelumab, which has demonstrated some positive results in T2-low patients, seems to be more efficacious in patients with T2-high inflammation [32,33]. The goals for severe asthma in the future are clear: on the one hand, we need to improve our biomarkers or combine them with new ones (even at the tissue level), which may not only lead to accurate prediction of the response to a biologic but also aid clinicians in the choice of the right mAb for patients eligible to receive more than one [34]; on the other hand, there still remain unmet needs for severe asthmatics who do not exhibit T2 inflammation, since this subgroup is currently not eligible for any biologic except for tezepelumab.

## 4. Chronic Obstructive Pulmonary Disease

In the context of COPD, despite the evolution of treatment options, the mainstay of therapy remains universal. The aim is maximum bronchodilation with the use of beta agonists and muscarinic receptor antagonists. The implementation of ICS in selected patients with a higher blood eosinophil count and frequent exacerbations is the first “personalized” step in COPD treatment [35,36]. Chronic inflammation in COPD differs from asthma, since it is mainly characterized by neutrophils, macrophages and CD8+ T-lymphocytes [37]. Inflammatory pathways are more difficult to target, and biomarkers are still lacking. This is part of the reason why advances in precision medicine for COPD are still in their infancy compared to those for asthma. Nevertheless, a T2-high phenotype can be present in COPD patients. Dupilumab (anti IL-4/13) is the first biologic to have received approval for COPD management in September 2024, paving the way for patient phenotyping [38,39]. Mepolizumab (anti-IL-5) is the second such biologic, having recently been approved for COPD treatment based on the results of a MATINEE trial [40], and although the comparison is indirect, dupilumab seems to have more consistent results in randomized controlled trials so far.

Other biologics hold promise for the future, since there are ongoing trials for benralizumab, tezepelumab, tozorakimab and itepekimab (anti-IL-33), and astegolimab (anti-ST2, acting as the receptor of IL-33) [41,42,43,44,45]. With regard to compounds associated with other mechanisms, the approved dual-phosphodiesterase 3/4 inhibitor ensifentrine represents another option in our arsenal, even if the exact population that could benefit from it has not been established yet and even though it is currently considered an add-on option [46,47,48]. Roflumilast (phosphodiesterase 4 inhibitor) is an additional therapeutic choice for frequent exacerbators with severe airflow limitation presenting with a chronic bronchitis phenotype. Due to tolerability concerns, this therapy is generally reserved for a minority of patients; however, in these individuals, it significantly reduces both the frequency of exacerbations and the need for hospitalization [49]. Other anti-inflammatory agents are in the pipeline of clinical trials as well, but so far, the results are not positive for a number of agents (Janus kinase inhibitors, p38 mitogen-activated protein kinase inhibitors, phosphatidylinositol 3-kinase inhibitors, nuclear factor-κB inhibitors, protease inhibitors and chemokine modifiers) [50,51]. Azithromycin is the only antibiotic that has demonstrated efficacy in COPD as a long-term treatment regimen for almost 15 years; this success is owed to the immunomodulatory, anti-inflammatory and antibacterial effects of macrolides. Ex-smokers with a high exacerbation risk are expected to benefit from the use of azythromycin, presenting with fewer exacerbations and an improvement in quality of life. Nevertheless, hearing decrements are common in the context of long-term use, and clinicians should remain vigilant [52].

Although precision medicine has been partially implemented in the phenotyping of stable disease, exacerbations of asthma and COPD still follow a “one-size fits all” approach. Studies from asthmatic populations underscore benralizumab’s unique feature of antibody-dependent cell mediated cytotoxicity, which results in a rapid depletion of eosinophils, as soon as 24 h after infusion. Benralizumab at a dose of 100 mg (more than 3 times higher than the regular dose in asthma) in patients with an eosinophilic (T2-high) exacerbation of asthma or COPD demonstrated extremely positive results in terms of reducing treatment failure compared to prednisone. The ABRA study paves the way for personalized management not only of stable obstructive lung diseases but also of acute exacerbations [53]. The key message is that the endotype of the disease could be more important than the actual name of the disease.

## 5. Bronchiectasis

Bronchiectasis has also become part of an intensive research effort to develop personalized treatment strategies. Neutrophilic and eosinophilic inflammation are the most clearly defined inflammatory subtypes. ICS are beneficial for the latter subtype, in a similar context to that of implementing ICS in COPD; however, more data are needed to adequately address this [54]. A fundamentally new approach directly targeting neutrophil serine protease activation is the administration of the recently approved brensocatib, a dipeptidyl peptidase-1 (DPP-1) inhibitor which prolongs the time to first exacerbation in patients with bronchiectasis [55]. Recently published European Respiratory Society guidelines for bronchiectasis underscore the clinical significance of a personalized approach based on the colonization of patients with *Pseudomonas aeruginosa*. In such cases, long-term inhaled antibiotics are suggested as an additional measure to prevent disease exacerbations. Long-term macrolides are also strongly recommended for patients with a high exacerbation risk [56]. Last but not least, the integration of microbiome analysis into the management of bronchiectasis and the treatment of “multibiome” as a whole rather than simply addressing individual pathogens has inspired a holistic approach considering microbial interactions, treating what we should treat and letting be what we should not tamper with [57].

## 6. Interstitial Lung Diseases

In the context of fibrotic ILDs, while substantial research progress has been made, little progress has been made with regard to the implementation of these findings in clinical practice [58,59,60]. For example, in terms of disease susceptibility, the polymorphism rs35705950 in the promoter region of mucin 5B (*MUC5B*) has the strongest link to Idiopathic Pulmonary Fibrosis (IPF) susceptibility, while short telomere length is also a risk factor [61]. Testing of telomere length is feasible in some centers; however, widespread clinical applicability is an unmet need. Accordingly, prognostic biomarkers, such as a telomere length less than the 10th percentile, the variant of *TOLLIP* (rs57438900) or the 52-gene signature, have not been implemented in clinical practice in most centers [58,62,63,64]. Accurate prognostication is very important for achieving timely interventions and optimizing the selection of ideal lung transplant candidates. The only clinically applicable biomarker that could be used towards this direction is monocyte count. Increased monocyte count has been associated with an increased risk of disease progression, hospitalization, and mortality in IPF [65,66,67], as well as with worse post-lung transplant survival. In terms of treatment response evaluation, a seminal study including two independent cohorts showed that patients with fibrotic hypersensitivity pneumonitis or unclassifiable ILD and leukocyte telomere length <10th percentile had reduced survival when exposed to mycophenolate mofetil or azathioprine [68]. Given that telomere length can predict disease susceptibility, prognosis and adverse events from immunosuppression, this biomarker might deserve widespread clinical implementation. Accordingly, evaluation of *TOLLIP* does not only have prognostic implications; *TOLLIP* TT genotype and CC genotype have been linked to better and worse response to N-Acetylcysteine (NAC), respectively [69]. This has led to the design of the PRECISIONS trial, a study investigating NAC response based on *TOLLIP* gene variants in IPF [70]. Moreover, analysis of the CleanUP-IPF study based on *TOLLIP* genotype demonstrated that doxycycline improved survival in patients with the TT genotype but was associated with worse survival in patients with the CC genotype [71]. This result is a typical paradigm of the importance of accurate endotyping. Similar studies have been performed to evaluate the response to antifibrotics, with mitochondrial DNA being among the biomarkers that hold promise [72]; nevertheless, further research is needed. Despite the aforementioned progress, therapeutic management is mainly defined by the presence or absence of progressive pulmonary fibrosis. While this framework can be beneficial in the current era of antifibrotic therapy, lumping together heterogeneous diseases under this umbrella impedes progress in understanding their distinct pathogenetic mechanisms and ultimately counteracts the principles of personalized medicine [73].

## 7. Future Perspectives and Concluding Remarks

Altogether, substantial progress has been achieved in the context of precision medicine approaches in chronic lung diseases. However, the roadmap to precision medicine still involves numerous stages. There is still a need for biologically enriched randomized controlled trials that are able to provide actionable information. An example of actionable information is a biomarker that can reliably predict which of the two treatment regimens is more effective in a particular endotype. Following the identification of this actionable information, there will still be infrastructural, financial, ethical and regulatory challenges to overcome for the implementation of precision medicine in clinical practice. Infrastructural challenges include the minimization of technical variabilities in the results of biomarker testing, so that results are reproducible across centers. Financial challenges include the cost of such approaches, while ethical challenges include the safety of personal data. Finally, in terms of regulations, there is always the ‘pacing issue’, given that scientific advances quickly outpace regulations and, thus, regulatory bodies should expedite related approvals. The scientific community bears responsibility to persuade the political community to advocate for cost-effective precision medicine. Precision medicine can limit cost incurred from the inappropriate use of expensive treatments and cost from hospitalizations that could be avoided with patient-centered management. If the aforementioned limitations are overcome, then a move from “one-size fits all” approach to personalized care will be more feasible. This could positively affect patients’ quality of life and survival.
